# Beyond essentiality: silicon as a systems regulator of photosynthesis under stress scenarios

**DOI:** 10.3389/fpls.2025.1690421

**Published:** 2026-01-09

**Authors:** Mohammad Mukarram, Andleeb Zehra, Shadma Afzal, Alena Sliacka Konôpková, Khalid Ali Khan, Abdulaziz R. Alqahtani, Haitham Ibrahim El-Mekkawy, Daniel Kurjak, Alexander Lux, Rizhao Chen, Qiyun Li

**Affiliations:** 1 College of Plant Protection, Jilin Agricultural University, Changchun, Jilin, China; 2 Advance Plant Physiology Section, Department of Botany, Aligarh Muslim University, Aligarh, India; 3 Department of Bioclimatology, Faculty of Environmental Engineering and Mechanical Engineering, Poznan University of Life Sciences, Poznań, Poland; 4 Department of Integrated Forest and Landscape Protection, Faculty of Forestry, Technical University in Zvolen, Zvolen, Slovakia; 5 Center of Bee Research, and its Products (CBRP), and Unit of Bee Research and Honey Production, King Khalid University, Abha, Saudi Arabia; 6 Applied College, King Khalid University, Abha, Saudi Arabia; 7 Department of Biology, College of Science, University of Bisha, Bisha, Saudi Arabia; 8 Department of Biology, Faculty of Science, King Khalid University, Abha, Saudi Arabia; 9 Institute of Forest Ecology, Slovak Academy of Sciences, Zvolen, Slovakia; 10 Department of Plant Physiology, Faculty of Natural Sciences, Comenius University in Bratislava, Bratislava, Slovakia

**Keywords:** silicon, photosynthesis, abiotic stress, omics, stomatal signaling, ABA, nitric oxide

## Abstract

Silicon (Si), although not classified as an essential element, has emerged as a key modulator of photosynthesis and stress resilience in higher plants. However, despite extensive reports on its beneficial effects, a clear mechanistic understanding of how Si modulates photosynthetic machinery under stressful environments remains fragmented and inconsistent. This review critically synthesises recent advances in Si-mediated regulation of photosynthesis under both optimal and stress conditions. We highlight its influence on chlorophyll stability, photosystem (PSII/PSI) efficiency, electron transport, stomatal conductance, and nutrient homeostasis. We emphasise Si’s interaction with phytohormones and signalling molecules, including abscisic acid (ABA), nitric oxide (NO), and reactive oxygen species (ROS), which integrate hormonal and redox regulation of guard cell function. Emerging multi-omics studies and silicon nanoparticles (SiNPs) reveal how Si alters transcriptional networks, protein stability, and metabolite balance to sustain photosynthetic performance. This review addresses the knowledge gap in connecting Si-driven nutrient regulation with photosynthetic resilience by bridging omics approaches, hormonal crosstalk, and nanotechnology interventions. We conclude that strategic Si supplementation can be a sustainable approach to strengthen plant photoproductivity under climate change scenarios.

## 1 Introduction

Photosynthesis is the cornerstone of plant productivity and global food security. However, climate change and associated abiotic stresses severely threaten this vital process. These stresses disrupt chloroplast integrity, electron transport chains, and chlorophyll content, compromising photosynthetic efficiency and crop yields. Without effective management, abiotic stresses could slash global crop yields by up to 50% in major food crops (Vogel et al., 2019). As agricultural systems grapple with these escalating pressures, innovative strategies to bolster photosynthesis for improved crop productivity are sought (Lawson et al., 2012; Croce et al., 2024).

**Figure 1:**
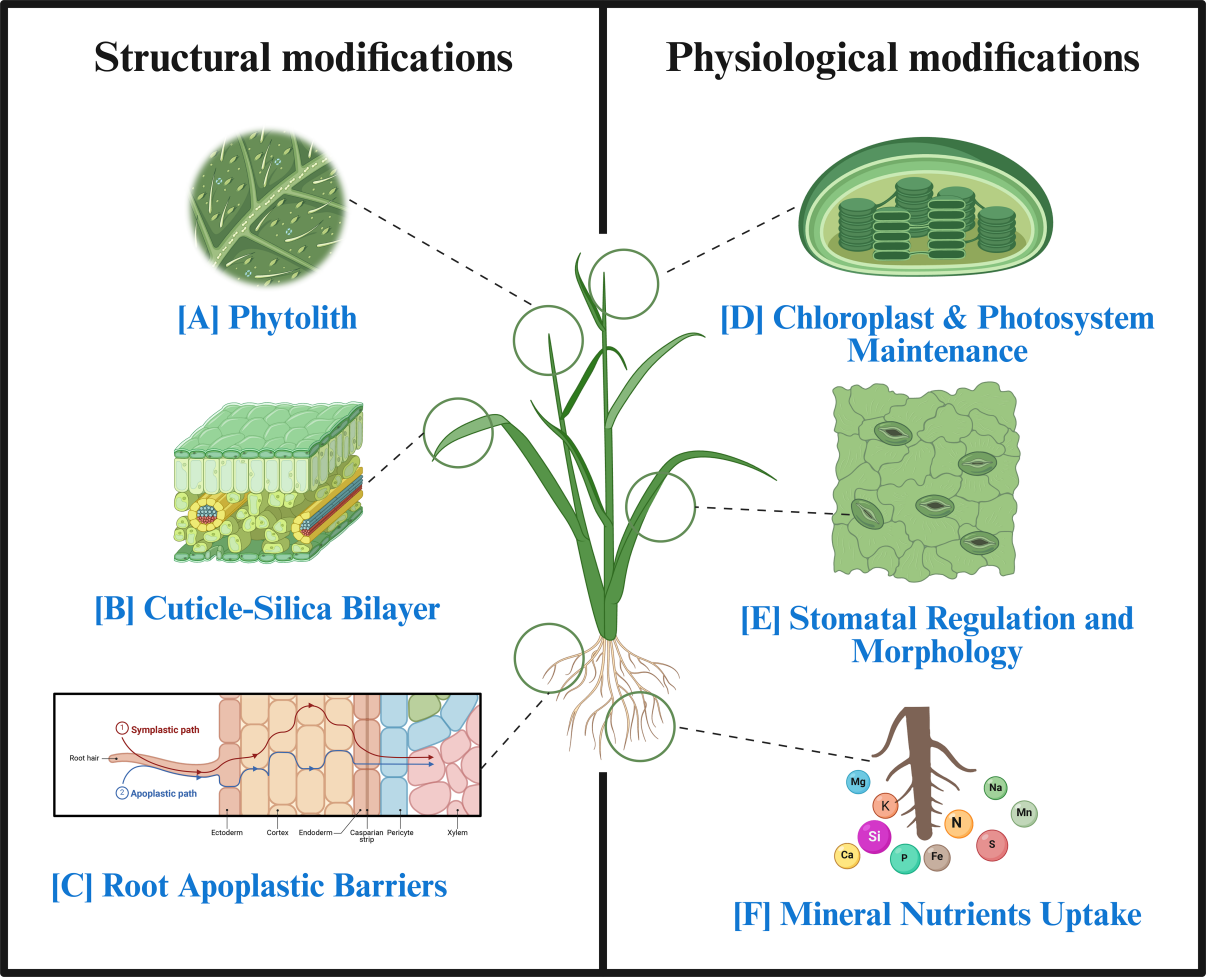
Photosynthesis-related upgradation by Si application. **(A)** Phytoliths: Si polymerises as amorphous silica in/between cell walls, making tissues harder to crush, more impact-absorbent, and less prone to fracture; this contributes to overall cell-wall fortification, and these specialised silica bodies are called ‘phytoliths’. **(B)** Cuticle-silica bilayer: Si often deposits beneath the epidermal cuticle. It helps in leaf thickening and erectness, canopy architecture, and lowering water loss. Overall, this contributes to improved photosynthesis by improving light interception and reducing self-shading. **(C)** Root apoplastic barriers: Si promotes endodermis/exodermis development and silicification. It reinforces Casparian/suberin bands and modifies ion movement. **(D)** Chloroplast and photosystem maintenance: Si mitigates oxidative stress, stabilises thylakoid membranes, and preserves chloroplast ultrastructure. It upregulates photosystem (PSII/PSI) proteins, chlorophyll fluorescence, and associated electron transport components. It sustains efficient light energy conversion and photochemical activity. **(E)** Stomatal regulation and morphology: Si can modulate guard-cell turgor, optimise stomatal aperture, and enhance stomatal density, size, or symmetry. This results in improved CO_2_ diffusion and water-use efficiency, maintaining a balanced stomatal conductance for photosynthesis. **(F)** Mineral nutrient uptake: Si enhances the uptake and translocation of essential nutrients such as N, P, Mg, Fe, and Mn. These improvements support chlorophyll synthesis, enzyme activation, and electron transport, ultimately reinforcing photosynthetic performance.

One such strategy is the application of silicon (Si) in agriculture. Si has been established as a quasi-essential element for several plants, particularly under stress contexts (Ahmed et al., 2023; Manivannan et al., 2023; Mitani-Ueno et al., 2023). Si confers multifaceted advantages, including structural reinforcement of cell walls, improved nutrient homeostasis, and augmented defence against environmental adversities (Ma and Yamaji, 2006; Ma et al., 2023) (**Figure 1**). Under optimal conditions, Si influences photosynthetic machinery by preserving thylakoid membrane stability, maintaining chlorophyll concentrations, and optimising gas exchange parameters (Song et al., 2014; Zhang et al., 2018). It promotes photochemical efficiency, quantum yield of photosystem II (PSII), and electron transport, while modulating gene expression related to photosynthetic proteins (Rastogi et al., 2021). Emerging research highlights Si’s broader utility in enhanced nutrient uptake via aquaporin regulation and root elongation, supporting overall photosynthetic capacity (Liu et al., 2015; Debona et al., 2017). During drought, Si improves leaf water potential, stomatal conductance, and antioxidant enzyme activities to combat reactive oxygen species (ROS)-induced chloroplast damage (Thorne et al., 2020; Shaw et al., 2025). Si reduces Na^+^ and Cl^-^ uptake in saline conditions, improves K^+^/Na^+^ ratios, and protects PSII reaction centres (Manivannan et al., 2016; Zhu et al., 2019). This supports net photosynthetic rates and pigment stability. Similarly, Si-mediated ion sequestration and compartmentalisation can mitigate metal(oid) toxicities and thylakoid disintegration (Vishwakarma et al., 2020; Zehra et al., 2020). Recent advances, including nano-silicon applications, have reported substantial photosynthetic adaptations under low-temperature and salt stresses by improving photochemical quenching and membrane stability (Soundararajan et al., 2014; Mukarram et al., 2023).

At the molecular level, omics technologies, i.e., transcriptomics, proteomics, and metabolomics, have unveiled Si’s regulatory networks, revealing upregulation of genes encoding photosynthetic components (e.g., RuBisCO, PSII subunits) and stress-responsive pathways (Muneer et al., 2014; Rastogi et al., 2020). Notably, Si interacts with stomatal signalling molecules like abscisic acid (ABA) and nitric oxide (NO), modulating guard cell responses to fine-tune transpiration and CO_2_ assimilation under stress (Mukarram et al., 2022b; Postiglione and Muday, 2020). This interplay enhances ROS homeostasis, prevents stomatal dysfunction, and promotes adaptive plasticity (Vandegeer et al., 2021). The present review synthesises the evolving narrative of Si’s role in photosynthesis across optimal and stress environments, drawing on insights from physiological, biochemical, and omics. We aim to underscore Si’s potential as a sustainable tool for crop improvement by elucidating its protective effects on the photosynthetic apparatus and synergies with signalling pathways.

## 2 Si under physiological conditions

The studies with Si treatment under an optimal environment are minimal, with even fewer studies reporting Si interaction with photosynthetic machinery. It is suggested that Si can regulate several aspects of photosynthesis, such as electron transport, chloroplast structure, photosynthesis-related genes, and the net photosynthetic rate (Lavinsky et al., 2016; Rastogi et al., 2020). This could stem from Si crosstalk with phytohormones, gaseous signalling molecules (e.g., H_2_O_2_), and nutrient absorption and transportation (Frew et al., 2018; Pavlovic et al., 2021). Conversely, some studies reported that Si does not affect chlorophyll content without stress (Zhang et al., 2018). However, in another study, foliar sprays of silicon nanoforms induced chlorophyll content, photochemical quenching, and chlorophyll fluorescence (Mukarram et al., 2021a). This suggests that silicon can have a differential impact on photosynthesis depending on its concentration, form, and mode of application (**Table 1**).

**Table 1:** Silicon interaction with photosynthetic machinery under physiological conditions.

Growth conditions	Plant species	Si source	Si size/ concentration	Mode of Si application	Effect	Reference
Physiological conditions	*Mentha piperita*	SiO_2_	50, 100, 150, and 200 mg L^−1^	Foliar spray	Increased content of chlorophylls, yield of chl a fluorescence, net photosynthetic rate, and content of phenols, essential oils, and menthol	Ahmad et al., 2020
*-*	*Triticum aestivum*	Na_2_SiO_3_	6 mM	Foliar spray	Enhanced chlorophyll *a*, *b*, and carotenoids content, chlorophyll and membrane stability indices, net CO_2_ uptake, and net assimilation rate	Maghsoudi et al., 2016
*-*	*Saccharum officinarum*	CaO·SiO_2_	0, 100, 300, and 500 mg L^−1^	Soil application	Improved photosynthesis, transpiration, and stomatal conductance, as well as stomatal density and stomatal aperture size	Verma et al., 2020a
*-*	*Salvia splendens*	K_2_SiO_3_	100 mg·L^-1^	Tosilee medium	Enhanced shoot and root growth and biomass, chlorophyll content, and antioxidant enzyme activity	Soundararajan et al., 2014
*-*	*Zea mays*	K_2_SiO_3_	45, 90, 150 and 225 kg ha^-1^	Soil application	Increased PSII photochemical efficiency, gas exchange, activity of antioxidant enzymes, and MDA content, contents of total soluble sugar and starch, growth, and yield in the highest Si levels	Xu et al., 2016
*-*	*Cucumis melo*	Na_2_SiO_3_	0, 0.5 and 1 mmol L^-1^	Nutrient solution	Boosted CO_2_ assimilation rate, nitrate depletion from the medium, and shoot biomass production	Neocleous, 2015

## 3 Si under stress conditions

While a stressful environment jeopardises both the structural and functional components, Si works antagonistically to this. For example, Si preserves chloroplast ultrastructure, thylakoid membranes, and PSII variables. It supports stomatal regulation, nutrient uptake, and redox homeostasis, thereby sustaining the core processes of light harvesting and CO_2_ fixation. Importantly, the precise mechanisms vary with stress type. It employs osmotic regulation during drought, ion exclusion under salinity, and detoxification under metal(oid) toxicity. Still, several convergent protective roles of Si are consistently observed. Here, we highlight how Si modulates photosynthetic performance under diverse abiotic stresses, including shared and stress-specific mechanisms (**Table 2**).

**Table 2:** Si interaction with photosynthetic machinery during stress environments.

Growth conditions	Specification of growth conditions	Plant species	Si source	Si size/concentration	Mode of Si application	Effect	Reference
Drought	10% (w/v) PEG-6000	*Solanum lycopersicum*	K_2_SiO_3_	2.5 mmol L^–1^	Hoagland solution	Enhanced content of assimilatory pigments and photochemical efficiency	Zhang et al., 2018
	15 days, soil water content 25.1%.	*Oryza sativa* L.	K_2_SiO_3_	1.5mM	Soil application	Increased photosynthetic rate, transpiration rate, and photochemical efficiency, and adjusted the mineral nutrient absorption.	Chen et al., 2011
	soil water potential -30 kPa and -60 kPa	*Solanum lycopersicum*	SiO_2_	1, 2, 3, and 4 g L^−1^	Foliar spray	Attenuated effect of water deficit on transpiration, stomatal conductance, and net photosynthetic rate	Morato de Moraes et al., 2020
	12 days, 50% of soil relative water content	*Triticum aestivum*	Na_2_SiO_3_	2.11 mmol	Soil application	Improved water status, net CO_2_ assimilation rate, contents of photosynthetic pigments, activity of the antioxidant system, contents of soluble proteins, and total thiols.	Gong et al., 2005
	60% and 40% field water capacity	*Triticum aestivum*	Na_2_SiO_3_	6 mM	Foliar spray	Improved leaf relative water content, gas exchange rate, chlorophyll *a*, *b*, and carotenoid content, chlorophyll stability index, and membrane stability index	Maghsoudi et al., 2016
Salinity and drought	6 g NaCl kg^-1^ + 45-50% or 30-35% FWC	*Glycyrrhiza uralensis*	K_2_SiO_3_	0.1g kg^-1^	Soil application	Improved chlorophyll content, net photosynthetic rate, and relative water content, growth rate, and dry mass	Zhang et al., 2020
Salinity	Saline water from Lake Urmia, Iran (0, 3, 5, 8, 10, 12, and 14 dS/m)	*Triticum aestivum*	K_2_SiO_3_	1.5 mM/4 mM	Seed soaking/foliar spray	Maintained the content of chlorophyll a and b, improved chlorophyll fluorescence parameters in lower salinity levels; however, the positive effects of Si application were lost in the highest salinity levels.	Feghhenabi et al., 2022
	160 and 240 mM NaCl	*Cymbopogon flexuosus*	SiNPs	150 mg L^-1^	Foliar spray	Enhanced growth, chlorophyll content, photosynthetic performance of PSII, gas exchange, antioxidant system, and consequently minimised oxidative stress markers, and improved content of essential oils	Mukarram et al., 2023
	75 mM or 150 mM of NaCl	*Capsicum annuum*	K_2_SiO_3_	2 mm	Hoagland nutrient solution	Improved photosynthesis, stomatal conductance, leaf water status, and membrane stability consequently led to enhanced growth and biomass production.	Altuntas et al., 2018
	250 mM of NaCl	*Solanum lycopersicum*	SiNPs	100 mg/L	Root dipping/foliar spray	Increased chlorophyll index, efficiency of chl a fluorescence, net photosynthetic rate, stomatal conductance and transpiration rate, internal CO2 concentration, activity of antioxidative enzymes, mineral content, and growth	Alam et al., 2022
Acidity	Simulated acid rain pH 4.0, 3.0, 2.0	*Oryza sativa*	Na_2_SiO_3_	1, 2, and 4 mM	IRRI nutrient solution	Improved ultrastructure of chloroplasts, chlorophyll content, maximal and actual quantum yield of PSII photochemistry, stomatal conductance, and growth	Ju et al., 2020
Heat	45 °C for 5 h	*Triticum aestivum*	K_2_SiO_3_/SiO_2_NPs	1.5, and 1.66 mM	Seed soaking	Increased photochemical performance of PSII, the photosynthetic pigments, and organic solutes, including soluble sugars, sucrose, and proline accumulation, attenuated electrolyte leakage	Hassan et al., 2021
	37 ± 2 °C for 30 days	*Triticum aestivum*	Not available	2, and 4 mM	Foliar spray	Boosted content of photosynthetic pigments, activities of enzymatic antioxidants, soluble sugar, protein, and proline, growth, and grain yield	Mustafa et al., 2021
	45 °C for 4 h	*Triticum aestivum*	K_2_SiO_3_/SiO_2_NPs	1.5, and 1.66 mM	Seed soaking	Enhanced photochemical performance of PSII, the content of photosynthetic pigments, restored ultrastructural distortions of the chloroplast nucleus, and membrane stability	Younis et al., 2020
Nutrient stress	P deficiency: 0, 0.01, and 0.1 mM P levels	*Triticum aestivum*	Na_2_SiO_3_	2 mM	Nutrient solution	Altered cell wall composition, which was associated with higher mesophyll conductance, net CO_2_ assimilation, and carbohydrate level	Ulloa et al., 2021
Metal(oid) stress	As: 3.2 mg L^−1^	*Solanum lycopersicum*	SiO_2_NPs	10–20 nm; 1000 mg L^−1^	Steiner solution	Increased chlorophyll content, antioxidant system capacity, modified nutrient uptake, and the bioactive compounds of tomato fruits	González-Moscoso et al., 2022
	Cd: 5 μM or 50 μM	*Zea mays*	Na_2_SiO_3_	5 mM	Hoagland solution	Boosted CO_2_ assimilation rate, effectiveness of PSII photochemistry, content of assimilation pigments, and alleviated distortion of thylakoid formation in chloroplasts	Vaculík et al., 2015
	Cd, Ni, Pb-contaminated soil (mg kg^-1^ soil): 18.2-18.6, 255-259, and 252-254, respectively	*Phaseolus vulgaris*	K_2_SiO_3_/ SiNPs	10 mmol L^−1^/2.5, and 5.0 mmol L^−1^	Foliar spray	Enhanced plant growth and production, content of assimilatory pigments, gas exchange parameters, membrane stability index, relative water content, free proline, total soluble sugars, the activities of the antioxidant system, and modified nutrient contents	El-Saadony et al., 2021
	Cd, Pb-contaminated soil (mg kg^-1^ soil): 2.82, and 300.06, respectively	*Triticum aestivum*	Fertiliser OSiFA/fertiliser OSiFB/Na_2_SiO_3_ ·9H_2_O	7.50 mg kg^−1^ /20 mg kg^−1^ / 8.5% Si	Soil application	Increased gas exchange, chlorophyll content, and grain yield diminished the oxidative damage, increased Si uptake in roots and shoots, reducing Cd and Pb accumulation, and thus decreased the health risk index of both Cd and Pb.	Huang et al., 2019
	Zn: 0, 25, and 50 μM	*Gossypium hirsutum*	Na_2_SiO_3_	0, and 1 mM	Hoagland solution	Suppressed Zn accumulation resulting in promoted biomass, photosynthetic performance, growth, and activity of the antioxidant system	Anwaar et al., 2015

### 3.1 Drought stress

Photosynthesis is one of the most vulnerable phenomena during drought owing to several stomatal and non-stomatal factors (Mukarram et al., 2021b). Many aspects of photosynthetic machinery can be restricted during drought, such as chlorophyll, pigment-protein complex, electron transport, chloroplast disintegration, and photosynthetic yield (Signarbieux and Feller, 2011; Qiao et al., 2024). Nonetheless, several reports argued that these photosynthetic constraints can be reversed with Si application (Waraich et al., 2011; Wang et al., 2019). Si improves water use efficiency and maintains higher leaf water content during drought by minimising leaf water loss and increasing root water uptake (Yin et al., 2014; Santos et al., 2023). Furthermore, Si-induced stomatal conductance, enzyme activities, net photosynthetic rate, and antioxidant metabolism defend against mounting ROS and chlorophyll degradation (Gong and Chen, 2012; Thorne et al., 2020). Similarly, exogenous Si treatment improved drought resistance in *Triticum aestivum* by increasing leaf water status, photosynthetic rate, and mineral nutrient absorption (Johnson et al., 2022; Chen et al., 2011). Similar results were seen in *Soghum bicolor*, *Hordeum vulgare*, and *Saccharum officinarum* (Hattori et al., 2005, 2007; Liu et al., 2014; Verma et al., 2020b).

Furthermore, Si can impact the uptake and translocation of several essential elements during drought. Si improved inorganic P content in several crops under drought conditions (Mückschel et al., 2025). A higher inorganic P content aids the ATP synthesis and further enriches CO_2_ assimilation. Additionally, Si increases polyamine biosynthesis during drought (Yin et al., 2014). This reflects positively on photosynthetic efficiency, considering polyamines support photosynthetic pigment content and delay leaf senescence.

### 3.2 Salinity stress

Salinity stress restricts physiology and biochemistry in higher plants, including chlorophyll functionality and overall photosynthetic efficiency (Mukarram et al., 2022a). Salt excess inhibits chlorophyll and carotenoid biosynthesis and RuBisCO content. Silicon, on the other hand, works antagonistically to salinity stress. It hampers the apoplastic transfer of Na^+^ and Cl^-^ (Shi et al., 2013). The development of double-cuticle layers by amorphous-Si lowers evapo-transpiration in Si accumulators. This can affect in two ways: (i) Si-mediated mechanical strength supports the photosynthetic canopy by improving the stiffness and erectness of leaves as well as reducing the self-shading (Soratto et al., 2012), (ii) reduced evapo-transpiration dilutes salt accumulation by maintaining higher plant-water status (Ali et al., 2012). The plant-water status and the transpiration rate can be regulated by the amount of Si gel connected with cellulose in the epidermal cell walls (Aziz and Gill, 2002). A similar finding observed that Si treatment reduced water loss in *Zea mays* by changing the morphological features of leaf epidermal cells (Ren et al., 2002). Several photosynthesis-related proteins, such as PSI, PSII, RuBisCo, and other chloroplast-related proteins, can be regulated by Si during salinity and hyperhydric conditions (Muneer et al., 2014; Soundararajan et al., 2017). Moreover, Si supplementation benefitted chlorophyll fluorescence and CO_2_ assimilation rate in *Cymbopogon flexuosus* (Mukarram et al., 2023). Si could have promoted the chloroplast size and the number of grana in leaves to confer such benefits (Yang, 2010).

### 3.3 Metal(oid) stress

The biological functions of proteins, lipids, and elemental components of thylakoid membranes are marginalised by metal(oid) toxicity. This results in stunted plant growth and productivity. For example, excessive Cu presence in the soil creates ROS-mediated cellular damage, inhibiting photosynthesis and delaying PS II repair (Gururani et al., 2015). However, the Si application can reverse Cu restrictions on photosynthetic machinery in *Oryza sativa* (Kim et al., 2014). This can be associated with the protection ability of Si to chlorophyll molecules (Vaculík et al., 2015). Furthermore, Cd toxicity can decrease photosynthesis through oxidative buildup, water scarcity, and mineral-uptake imbalance (Metwally et al., 2005; Farooq et al., 2013). Nonetheless, Si supplementation minimises Cd-induced oxidative burst by quenching H_2_O_2_ and O_2_^•-^ and relieves photosynthetic constraints (Hasanuzzaman et al., 2017). Similarly, Cr can restrict photosynthetic assembly through several ultrastructural changes, such as unequal swelling of the chloroplast, increasing amounts of plastoglobuli, disintegration and disappearance of thylakoid membranes, and an increase in the size and number of starch granules in leaf mesophyll cells (Huda et al., 2017). However, such photosynthetic constraints were mitigated with Si supplementation (Ali et al., 2012; Huda et al., 2017). It is suggested that Si mediates this feedback mechanism through palliating metal uptake, cellular compartmentalisation, and activating antioxidative enzymes (Mukarram et al., 2024). Zn, on the other hand, is an essential micronutrient for optimal plant growth. It is engaged in mitochondrial respiration, electron transport, superoxide scavenging, lignification of cell walls, and others (Stanton et al., 2022). While Zn deficit can reduce chlorophyll content, net photosynthetic rate, and superoxide dismutase activity, excess Zn causes leaf chlorosis and impaired photosynthesis (Kaur and Garg, 2021). This can be overcome by Si application, considering silicon can alleviate Zn phytotoxicity. Si can cut Zn transit from roots to shoots in *Oryza sativa* and expand Zn binding to the cell wall (Vaculík et al., 2012).

### 3.4 Temperature stress

High temperatures can hamper the photosynthetic machinery through photorespiration and disruption of chlorophyll biosynthesis (Ding and Yang, 2022). Similarly, chilling stress has been reported to negatively influence photosynthesis in several crops (Gusain et al., 2023). Hu et al. (2020) discovered that Si preserved stomatal opening in *Euphorbia pulcherrima* during high-temperature stress. Increased stomatal opening leads to higher evaporation, which reduces the risk of thermal injury to tissues through leaf cooling (Crawford et al., 2012). Furthermore, Si was also reported to promote epicuticular wax deposition in banana (Asmar et al., 2013) and strawberry (Braga et al., 2009) plants. Epicuticular wax regulates stomatal conductance, reflects irradiance, and lowers water loss (Huggins et al., 2018). Furthermore, Si-treated plants exhibit a higher chlorophyll fluorescence (F_v_/F_m_) and preserve more photosynthetic proteins (Muneer et al., 2017; Hu et al., 2020). This signifies Si as a potent elicitor for the photosynthetic apparatus under temperature stress.

## 4 Si and mineral nutrient interactions

Silicon modulates plant nutrient dynamics by enhancing the uptake and distribution of key macro- and micronutrients vital for photosynthesis (Greger et al., 2018; Pavlovic et al., 2021; Mückschel et al., 2025). Elements such as Mg, Fe, Cu, and Mn are directly involved in chlorophyll synthesis, electron transport, and water-splitting, while P, K, and Na regulate ATP formation and carbon assimilation. These nutrients collectively sustain efficient energy conversion and assimilate transport in plants. Liu et al. (2015) reported that Si could increase nutrient uptake by stimulating aquaporin genes and promoting root elongation activity. Similarly, Si fertilisers assist in water uptake, transport, and ion homeostasis by enhancing photosynthesis (Debona et al., 2017; Schaller et al., 2023, 2024). Trace elements (TEs)-induced stress disrupts the uptake and accumulation of several essential micronutrients (B, Mn, Fe, and Zn) and macronutrients (Ca, N, S, P, and Mg) (Mukarram et al., 2024). Nonetheless, Si application increased the nutrient content in several crops, including fava bean (N, P, Ca), aloe (P, Ca, Mg), tomato (P, Ca, Mg), canola (P, Fe), and cucumber (Ca) (reviewed by Rastogi et al., 2021). Recent evidence further reinforces these interactions: Si-mediated improvements in rhizosphere nutrient bioavailability and organic acid exudation enhanced P and Fe mobility in soil-plant systems (Chibesa et al., 2025). Likewise, Si application has been linked to improved coupling between soil physicochemical traits and nutrient uptake efficiency, especially under stress conditions, highlighting its pivotal role as a biogeochemical modulator in sustainable nutrient cycling (Bokor et al., 2021; Hodson and Guppy, 2022; de Tombeur et al., 2024; Monoshyn et al., 2025).

## 5 Si and ABA-mediated stomatal signalling

ABA is a central root-to-shoot messenger regulating stomatal responses. It typically accumulates in response to abiotic stresses such as salt, cold, and drought to induce stomatal closure to conserve water (Mukarram et al., 2021b). Parallely, it modulates root system architecture, transcriptional and post-transcriptional gene expression, and metabolic networks to promote osmotic adjustment in leaf tissues (Bharath et al., 2021; Aslam et al., 2022; Lim et al., 2015). Stomatal regulation also involves a complex network of secondary messengers (ROS, NO, and Ca^2+^), and phytohormones [jasmonic acid (JA) and salicylic acid (SA)] under stress conditions to activate ion channels in guard cells, causing water loss and stomatal closure (Prodhan et al., 2018; Mukarram et al., 2021b; Bharath et al., 2021).

However, the effect of Si on the stomatal signalling pathway remains inconsistent across studies. For instance, Lee et al. (2010) reported a decline in ABA content in Si-treated soybean seedlings under salinity stress. Another study indicated that Si-induced reductions in ABA homeostasis were associated with increased levels of ABA degradation products (phaseic acid and dihydrophaseic acid) (Hosseini et al., 2017). It indicates that Si promotes ABA breakdown under stress conditions, potentially helping the plant to modulate its stress response more efficiently. Consistent with these findings, Gao et al. (2022) demonstrated that Si pretreatment restricted ABA biosynthesis during drought. It resulted in more lateral root growth, water uptake, stomatal opening, and improved photosynthetic efficiency. Si also regulated hormonal balance by increasing levels of cytokinins (CKs), gibberellins (GAs), and auxins (IAAs) to suppress drought effects through coordinated hormonal signalling and enhanced metabolic activity.

Despite these findings, other studies highlight the context-dependence of the Si-ABA interaction, varying by applied Si concentration (Koentjoro et al., 2021) and time elapsed since treatment (Xu et al., 2017). ABA levels rose significantly at 6 and 12 hours but returned to baseline after 24 hours in short-term studies with Si under salinity stress. This was attributed to the transient upregulation of NCED1 and NCED4 genes, followed by their downregulation (Kim et al., 2017). Similarly, Si enhanced salt tolerance in tobacco by increasing *NtNCED1* and *NtNCED5* expression while suppressing the ABA catabolism gene *NtCYP707A*. The resulting ABA accumulation activated aquaporin gene expression, improved root hydraulic conductivity, and maintained leaf water content (Liu et al., 2024). Kim et al. (2014) showed that Si initially reduced ABA levels in rice under Cd/Cu stress, but were significantly elevated by day 10. This suggests that Si may initially mitigate the plant’s stress response, but ABA-related signalling becomes progressively intensified as the stress persists. Further, Chen et al. (2024) showed that Si suppressed SA biosynthesis during fungal attack while upregulating ABA- and JA-related defence genes, as well as antifungal metabolites like chlorogenic acid and lignin.

These findings suggest that Si modulates ABA biosynthesis and amplifies its downstream signalling effects. This includes the enhancement of secondary messengers such as ROS, Ca^2+^, NO, and RNS, all of which mediate ABA-triggered stomatal closure (Hancock et al., 2011; Gong et al., 2020). ABA induces ROS in guard cells, where these molecules activate ion channels that lead to turgor loss and stomatal closure. Si further boosts ROS production while regulating antioxidant activity, helping prevent oxidative damage (Postiglione and Muday, 2020; Ranjan et al., 2021). Furthermore, Si increases guard cell sensitivity to ABA, as evidenced in fescue plants, where Si-treated individuals exhibited stronger stomatal closure at equivalent ABA levels (Vandegeer et al., 2021).

## 6 Shared mechanisms of Si-mediated photosynthetic protection

**Figure 2:**
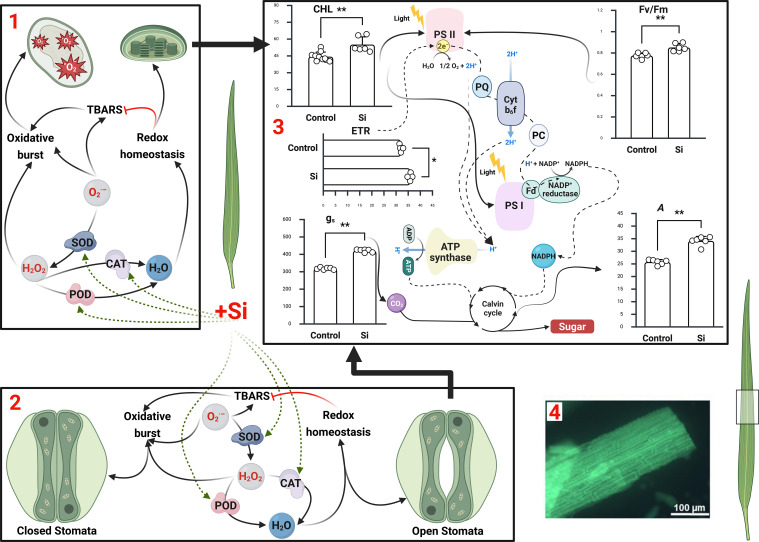
Integrative model of silicon-mediated enhancement of photosynthesis through redox balance, stomatal regulation, and photochemical efficiency. Silicon (Si) supplementation mitigates oxidative stress and stabilises photosynthetic function across cellular, stomatal, and chloroplastic scales. **(A)** Under stress, excess reactive oxygen species (ROS) such as superoxide (_2_^•–^) and hydrogen peroxide (H_2_O_2_) disrupt chloroplast integrity and electron transport. Si enhances antioxidant enzyme activities (SOD, CAT, POD), reducing ROS accumulation and restoring redox homeostasis. **(B)** In guard cells, Si modulates ABA- and ROS-dependent signalling in guard cells, maintaining stomatal aperture and CO_2_ flux for sustained photosynthetic activity. **(C)** Within chloroplasts, Si preserves PSII and PSI integrity. Si supports efficient electron transport rate (ETR), photochemical efficiency (Fv/Fm), chlorophyll levels (CHL), and gas-exchange parameters (assimilation rate *A* and stomatal conductance gs), reflected in the bar graphs. Each bar represents mean+SD. Values are expressed as follows: CHL, mol m-2 (SPAD values); g_s_, mmol CO_2_ m^-2^ s^-1^; *A*, μmol CO_2_ m^-2^ s^-1^. All bar graph data were analysed using the Mann–Whitney U test (non-parametric comparison between control and Si-treated samples) with significance accepted at p < 0.05 unless otherwise stated. p-values (two-tailed, approximate): ≤ 0.05, *≤ 0.005. Reproduced from Mukarram et al. (2023) and other lab data. **(D)** Microscopic image showing silica deposition beneath the leaf epidermis, forming a structural barrier that enhances tissue rigidity and photoprotective potential. Reproduced from Guerriero et al., 2020. Collectively, Si acts as a systemic regulator linking redox homeostasis, stomatal signalling, and photochemical stability to optimise photosynthetic performance under abiotic stress.

Across diverse abiotic stresses, several universal mechanisms emerge as the foundation of Si-mediated enhancement of photosynthesis. First, Si consistently improves ROS detoxification by stimulating antioxidant enzymes (SOD, CAT, APX, GR), thereby reducing oxidative damage to chloroplast membranes and photosynthetic proteins (Mukarram et al., 2022b) (**Figure 2**). Second, it preserves chlorophyll pigments and thylakoid integrity, delaying senescence and maintaining light-harvesting efficiency. Third, Si optimises nutrient homeostasis, particularly for K, Mg, Fe, and P, which are essential for ATP synthesis, chlorophyll biosynthesis, and electron transport (Pavlovic et al., 2021; Mukarram et al., 2024). Structural reinforcement through silica deposition further enhances leaf erectness and reduces transpirational water loss, indirectly supporting photosynthesis under stress. These cross-cutting benefits are complemented by stress-specific mechanisms, such as reduced Na^+^ uptake under salinity, metal detoxification under metal(oid) exposure, and stomatal cooling under heat stress.

Additionally, Si regulates stomatal behaviour, which balances CO_2_ uptake with transpirational water loss. Si interacts with ABA, NO, Ca^2+^, and reactive oxygen/nitrogen species (ROS/RNS) in guard cells. Depending on the stress context, Si may either suppress or promote ABA accumulation, thereby fine-tuning stomatal closure and water-use efficiency. Si also increases guard cell sensitivity to ABA and enhances secondary messenger signalling (ROS, NO, Ca^2+^), improving stomatal control under abiotic stress (Mukarram et al., 2022b). This dual role, modulating both ABA biosynthesis and its downstream signalling, makes Si a critical integrator of hormonal and redox networks that directly impact photosynthetic efficiency (**Figure 2**).

Together, these mechanisms suggest that Si functions as a universal stabiliser of photosynthetic machinery and a context-dependent regulator that tailors plant responses to particular stress conditions. This dual role is increasingly evident in omics studies, where Si influences the expression of genes, proteins, and metabolites linked to chloroplast function, antioxidant defence, and hormonal signalling.

## 7 Multi-omics insights into Si-mediated photosynthetic resilience

Multi-omics investigations attempt to interpret the *cause* of Si’s protective *effects* on photosynthesis. Each layer provides complementary insights:

- Genomics identifies Si-responsive genes, including transporters, aquaporins, heat-shock proteins, and transcription factors that regulate stress tolerance.
- Transcriptomics reveals changes in gene expression under stress, including modulation of SOS, NHX, and ABA-related pathways that directly impact ion balance and stomatal control.
- Proteomics can capture shifts in PSI/PSII proteins, RuBisCO, and antioxidant enzymes, linking Si to stabilising chloroplast function.
- Metabolomics and ionomics highlight Si-induced accumulation of antioxidants, amino acids, and osmolytes, and fine-tuning of mineral nutrient homeostasis critical for photosynthesis.

These insights attempt to unravel how Si not only alleviates specific stress-induced damages and reprograms photosynthetic machinery through multi-level regulation. Here, we provide a molecular framework for Si-mediated stress tolerance through a multi-omics approach.

### 7.1 Genomic insights

Silicon enhances photosynthetic resilience under stress by upregulating key PSI and PSII genes involved in light harvesting, water splitting, and electron transport. It induces genes such as *PsbB, PsbD, PsbH, PsbY, PsaH, PetC, PetH, PetE*, and *PetF*, which sustain chloroplast integrity and photochemical efficiency (Etienne et al., 2021). Collectively, Si-mediated transcriptional regulation stabilises the photosystems and preserves photosynthetic performance during abiotic stress. Moreover, aquaporin genes are upregulated by Si, which improves water transport and hydraulic conductivity under drought and salinity (Manivannan and Ahn, 2017; Manivannan et al., 2016). Similarly, Si upregulates the expression of heat shock transcription factors (Hsf), resulting in increased heat shock protein (HSP) synthesis in *Solanum lycopersicum* (Khan et al., 2020). Similar results were reported in European beach trees where Si application increased expression of hsp70 and hsp90 across all provenances (Nowakowska et al., 2024). Si treatment upregulated *AREB* (ABA-responsive element-binding protein), *CRK1* (cysteine-rich receptor-like protein kinase 1), and *TAS14* (ABA- and environmental stress-inducible) genes under salt stress, indicating activation of ABA-dependent signalling (Almutairi, 2016). Si was also reported to activate the glutathione reductase gene (*LeGR/SlGR*) in mitigating stress constraints in *Solanum lycopersicum* (Kaushik and Saini, 2019). Similarly, Si enhances the expression of late embryogenesis abundant (LEA) proteins, NAC-domain transcription factors, and metallothioneins, which collectively stabilise proteins, delay senescence, and to improve ion detoxification (Takasaki et al., 2010; Hussain et al., 2011; Khattab et al., 2014; Kaushik and Saini, 2019). These gene-level responses provide a foundation for sustaining chloroplast stability and PSI/PSII activity during stress.

### 7.2 Transcriptomic insights

Building on these gene-level findings, transcriptomics provides broader insight into how Si fine-tunes ion transport and signalling pathways linked to photosynthesis. Si supplementation alters the expression of SOS1/2, HKT, and NHX transporters during salinity, improving Na^+^ exclusion and K^+^ retention (Bosnić et al., 2018). Si regulated several genes (1237↑ and 232↓) in *Cucumis sativus*, several connected to plant metabolism, signalling, and ion homeostasis (Zhu et al., 2019). Other transcriptomic changes involve ABA- and ROS-regulated genes, suggesting that Si coordinates hormonal and redox signalling that directly impacts stomatal behaviour and CO_2_ assimilation (Mukarram et al., 2022b). Notably, Si can partially restore stress-altered transcriptomes toward control-like patterns, underscoring its regulatory rather than purely protective role (Chain et al., 2009).

### 7.3 Proteomic insights

Extending from transcriptional regulation to protein function, proteomic studies highlighted how Si safeguards the structure and efficiency of the photosynthetic apparatus. Si improved the expression of chloroplast proteome under salinity stress, offering better regulation of stomatal conductance, photosynthetic efficiency, and transpiration (Muneer et al., 2014; Muneer and Jeong, 2015). Si also enhances the abundance of ubiquitin ligases, chaperones, and protein-folding machinery, which protect against misfolded proteins in chloroplasts (Yang et al., 2006; Manivannan et al., 2016). Moreover, Si treatment improved photosynthetic efficiency by controlling LYP9 thylakoid membrane protein in *Oryza sativa* under drought (Wang et al., 2019). Furthermore, Si increased chaperone proteins like ClpC3, which decreases protein denaturing in chloroplasts. These adjustments confirm that Si protects photosynthetic machinery from oxidative stress and facilitates the repair and turnover of proteins essential for energy capture and carbon fixation.

### 7.4 Metabolomic and ionomic insights

Complementing these proteomic observations, metabolomic and ionomic studies provide an integrated view of Si’s downstream effects on metabolism and nutrient balance. At the metabolite level, Si induces the accumulation of antioxidants, osmolytes, and amino acids, all of which mitigate oxidative and osmotic stress (Mukarram et al., 2022b). Metabolomic investigations revealed that Si promotes the remobilisation of amino acids in *Oryza sativa* (Detmann et al., 2012). Si promotes metabolites, such as glutathione, ascorbate, flavonoids, tocopherol, and phenol, to palliate oxidative and osmotic burst (Metwally et al., 2018). Several other studies reported that Si boosts the contents of carotene, anthocyanin, succinate, leucine, proline, histamine, cysteine, and glutamic acid (Habibi, 2016; Cao et al., 2017; Kaushik and Saini, 2019). These energy-related metabolites and structural amino acids support chloroplast metabolism and the photosynthetic apparatus. Antioxidants such as boldine, myristic acid, allithiamine, pyridoxine, and cepharanthine are also promoted with Si supplementation. Complementary ionomic studies reveal that Si improves the uptake and distribution of K, Mg, Fe, P, and Ca, while reducing toxic ions such as Na^+^ and Cd^2+^ (Pavlovic et al., 2021). These shifts safeguard chlorophyll biosynthesis, electron transport, and ATP production, ultimately reinforcing photosynthetic resilience under adverse environments.

### 7.5 Integrated perspective

**Figure 3:**
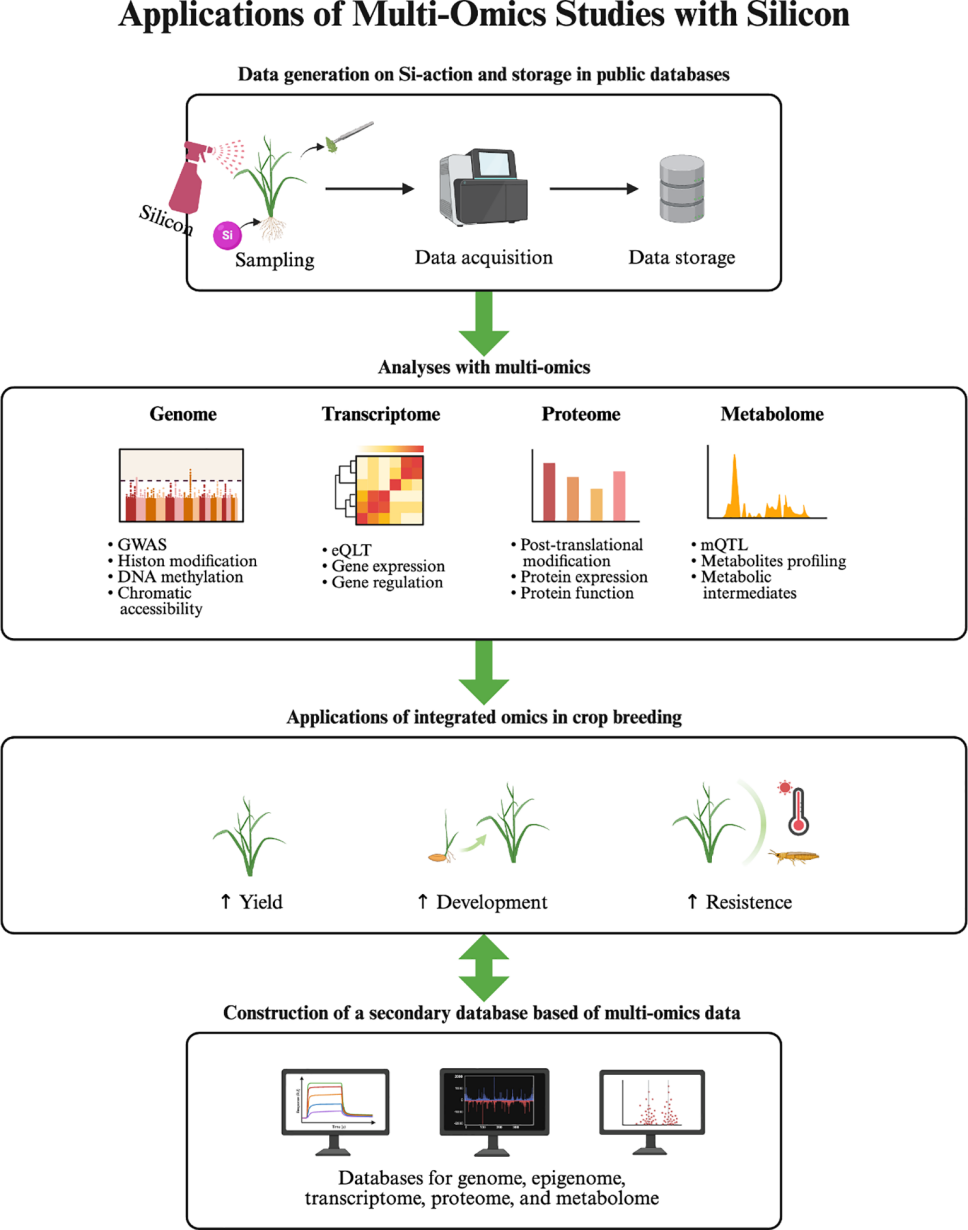
Conceptual workflow of multi-omics applications in silicon research for photosynthetic improvement. This schematic illustrates the sequential integration of omics-based approaches to understand and exploit silicon (Si)-mediated photosynthetic improvement in plants. Firstly, experimental data on Si-photosynthesis action are generated from plants grown under controlled and stressed environments and uploaded to public repositories for global accessibility. Secondly, multi-omics analyses, including transcriptomics, proteomics, metabolomics, and ionomics, can identify Si-responsive genes, proteins, and metabolites that underpin photosynthesis-associated regulation. Thirdly, integrated omics insights can be applied in crop breeding to select Si-efficient genotypes exhibiting improved photosynthesis, yield, and tolerance to extreme environments. Lastly, integrating multi-omics datasets can support the construction of secondary databases and predictive computational models for Si-responsive networks. These steps outline a systems-biology pipeline linking molecular discovery to translational breeding and digital data integration in Si-mediated photosynthetic improvements.

Multi-omics studies demonstrate that Si does not act through a single pathway but reprograms plants at multiple regulatory levels, from gene expression to protein stability, metabolite accumulation, and ion balance. This systemic regulation aligns with physiological observations that Si simultaneously preserves chloroplast ultrastructure, optimises stomatal behaviour, and enhances redox homeostasis. Furthermore, identification of potential molecular markers with omics could guide breeding and biotechnological interventions to improve Si use efficiency in crops. Therefore, multi-omics approaches are gaining attention for an elaborated understanding of several key physiological phenomena across species (Gupta et al., 2023; Ahmed et al., 2024; Hina et al., 2024) (**Figure 3**).

### 7.6 Gaps in the multi-omics approach

Despite the advances in multi-omics approaches, several critical limitations remain underexplored. For instance, MYB transcription factor family emerged as a Si-responsive candidate in wheat via transcriptomics; however, its functional role and expression patterns are missing in several other crops (Hao et al., 2021). Considering Si response can be genotype-specific and stress-dependent, cross-species validation of such molecular markers is rare (Cukrov et al., 2025; Shaw et al., 2025). Moreover, we still have limited meta-analysis and cross-study comparisons due to methodological heterogeneity with Si studies (e.g., size, concentrations, growing conditions, sampling times) (Cooke and Leishman, 2016; Fan et al., 2022). Multi-omics integration faces several computational challenges (data heterogeneity, missing values, collinearity, and noise) that make cross-layer correlation and network modelling difficult (López de Maturana et al., 2019; Morabito et al., 2025). This indicates that some proteins responsive to Si treatment might not be paralleled by transcript changes and *vice versa*.

## 8 Recent interventions with silicon nanoparticles

Conventional Si fertilisation has well-documented benefits, yet the emergence of silicon nanoparticles (SiNPs) offers new opportunities for enhancing photosynthetic resilience. It is suggested that due to their smaller size, SiNPs movement across cell membranes is more rapid and can be absorbed quickly. This increases silicon content inside the plant that manifests a more intense effect. Empirical studies have shown that SiNPs application improves chlorophyll content, chlorophyll fluorescence, photochemical quenching, and electron transport rate under optimal conditions (Ahmad et al., 2020; Mukarram et al., 2021a; Li et al., 2023; Kumari et al., 2024). Similar upregulatory effects on the photochemistry were seen when the plants were exposed to salinity and low-temperature stress (Liang et al., 2023; Mukarram et al., 2023; Li et al., 2025). Eghlima et al. (2024) also reported that nano-Si foliar application improved drought tolerance in *Calendula officinalis* L. by raising ABA. ABA is crucial in maintaining stomatal regulation under drought. However, we still need more studies to validate the direct involvement of SiNPs in ABA-associated stomatal regulations. During metal(oid) toxicity, SiNPs reduce metal content in aboveground plant tissues through compartmentalisation, metal immobilisation, or sequestration. This, along with improved stomatal regulation, assuages metal-induced oxidative bursts in the chloroplast and releases photosynthetic restriction (Fatemi et al., 2020; Mukarram et al., 2024; Pietrzak et al., 2025). Similar photoprotective effects of SiNPs application were observed during UV-B stress in *Solanum lycopersicum* (Copaciu et al., 2025).

Despite these promising results, key questions remain unresolved. The precise uptake pathways, subcellular localisation, and long-term safety of SiNPs are still under investigation (Mukarram et al., 2025). Moreover, variability in particle size, coating, and concentration makes establishing universal protocols for field use difficult. Nevertheless, the consistency of positive outcomes suggests that SiNPs represent a next-generation strategy for targeted Si delivery. This has strong potential for applications in precision agriculture and climate-resilient crop production. By bridging classical plant nutrition with nanotechnology, SiNPs highlight the future trajectory of Si research. This motivates researchers from descriptive evidence of stress mitigation toward a mechanistic understanding and practical innovation. This positions Si as a quasi-essential nutrient and a scalable biostimulant that can be optimised for sustainable agriculture.

## 9 Conclusion and future perspectives

Silicon influences photosynthesis through three interlinked modes of action: (i) biochemical interaction with photosynthetic machinery (e.g., PSII stabilisation and repair), (ii) mechanical reinforcement via silica deposition that alters leaf architecture and gas exchange, and (iii) transcriptional and metabolic reprogramming revealed by genomics, transcriptomics, proteomics, and metabolomics (Frew et al., 2018; Etienne et al., 2021; Lesharadevi et al., 2021). Across stresses such as drought, salinity, metal(oid)s, and temperature extremes, Si consistently enhances ROS detoxification, nutrient homeostasis, and chlorophyll stability. Furthermore, its regulatory interplay with stomatal signalling molecules (ABA, NO, ROS, Ca^2+^) fine-tunes photosynthetic efficiency under dynamic environments (Mukarram et al., 2022b).

Despite extensive evidence, essential questions remain unresolved:

1. Are Si-mediated benefits on photosynthesis predominantly direct (chloroplast-level) or indirect (via stress mitigation and signalling)?
2. How does Si dynamically interact with ABA and other hormones across different stress timelines?
3. Can omics approaches help identify universal “Si-responsive markers” that predict photosynthetic resilience?
4. To what extent can SiNPs replace or complement conventional Si fertilisation in field conditions?

Future research should integrate multi-omics datasets with high-resolution imaging and physiological assays to clarify the mechanistic basis of Si-photosynthesis interactions. Combining Si supplementation with genetic engineering of Si transporters and targeted use of SiNPs holds promise for developing climate-resilient crops. By bridging fundamental mechanisms with applied agronomy, Si can be strategically leveraged as a sustainable tool to safeguard photosynthesis and productivity in a changing world.
